# Foodborne Botulism Outbreaks in the United States, 2001–2017

**DOI:** 10.3389/fmicb.2021.713101

**Published:** 2021-07-16

**Authors:** Carolina Lúquez, Leslie Edwards, Chelsey Griffin, Jeremy Sobel

**Affiliations:** Centers for Disease Control and Prevention, Atlanta, GA, United States

**Keywords:** *Clostridium botulinum*, foodborne botulism, botulism outbreak, epidemiology, public health

## Abstract

Foodborne botulism is an intoxication caused by ingestion of food containing botulinum neurotoxin. Cases of foodborne botulism are usually sporadic (single, unrelated) but outbreaks of two or more cases occur. In this mini-review we will examine the following for the period 2001–2017, in the United States: botulism surveillance data, outbreaks of botulism affecting 10 or more people, and the public health preparedness and response approach.

## Introduction

Botulism is a rare, potentially fatal illness caused by botulinum neurotoxins (BoNT), produced by *Clostridium botulinum*, and rare strains of *C. butyricum* and *C. baratii* (Sobel, 2005). Seven antigenically distinct BoNTs have been identified, denominated serotypes A, B, C, D, E, F, and G (Sobel, 2005). Serotypes A, B, E, and F cause botulism cases in humans. Botulism is characterized by cranial nerve palsies which may be followed by flaccid, symmetrically descending paralysis. Four forms of naturally occurring botulism have been described: foodborne botulism, infant botulism, wound botulism, and adult intestinal colonization. Foodborne botulism is caused by the ingestion of foods contaminated with BoNT. Wound botulism is caused by spores of BoNT-producing species of *Clostridium* that germinate in a contaminated wound; the vegetative cells then multiply and produce BoNT *in situ*. Infant botulism is caused by BoNT-producing species of *Clostridium* that colonize the intestinal tract of infants (children under 1 year of age). Adult intestinal colonization is similar to infant botulism, but it affects persons older than 1 year (Sobel, 2005).

## Epidemiology of Foodborne Botulism in the United States, 2001–2017

According to CDC’s National Botulism Surveillance, from 2001 to 2017, 326 laboratory confirmed foodborne botulism cases were reported in the United States (Table 1; CDC, 2017). The median number of confirmed cases per year was 19 (range: 2 to 39 cases). In 2013, a case definition for additional probable foodborne botulism was implemented by the Council of State and Territorial Epidemiologists (CSTE) that includes patients whose clinical presentation is compatible with botulism and who had an epidemiological risk factor, such as consumption of home-canned food in the 48 h preceding illness onset (CDC, 2011). From 2013 to 2017, 29 probable foodborne botulism cases were identified in the United States and the median number of cases per year was 6 (range: 2 to 8 cases).

**TABLE 1:** Laboratory confirmed foodborne botulism cases in the United States, 2001–2017.

Year	Type A	Type B	Type E	Type F	NI^(1)^	Total
2001	20	2	10	1	0	33
2002	5	2	14	0	0	21
2003	5	0	2	1	0	8
2004	12	0	2	0	0	14
2005	7	1	10	0	0	18
2006	12	1	1	0	5	19
2007	15	4	7	0	0	26
2008	10	0	6	0	2	18
2009	10	1	0	0	0	11
2010	3	3	2	0	1	9
2011	14	0	5	1	0	20
2012	19	3	3	0	0	25
2013	1	0	1	0	0	2
2014	4	4	7	0	0	15
2015	34	0	5	0	0	39
2016	25	1	3	0	0	29
2017	15	0	4	0	0	19
TOTAL	211	22	82	3	8	326

*^(1)^Serotype not identified.*

The median age of patients with laboratory confirmed cases was 49 years (range: 6 months to 92 years). Most cases occurred in males (183 cases, 56%). Death was reported in 17 cases (5% case fatality ratio) and the median age of patients who died was 76 years (range: 53 to 91 years).

**FIGURE 1:**
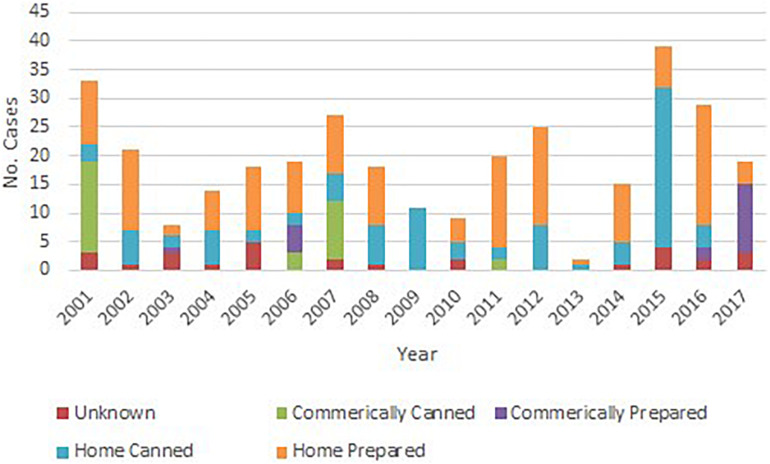
Number of foodborne botulism cases by year and type of food preparation, United States, 2001–2017.

A food or beverage was implicated in the epidemiological investigation for 277 (85%) laboratory confirmed botulism cases, and a food or beverage sample was laboratory confirmed as the source of BoNT in 156 (47%) cases. The method of food preparation implicated among the cases included foods prepared at home other than home canned foods (154 cases, 47%), home canned foods (94 cases, 29%), commercially canned foods (31 cases, 10%) and commercially prepared foods (20 cases, 6%) (Figure 1). The method of food preparation was not available for 27 (8%) cases. Pruno, an illicit prison-brewed alcoholic beverage (45 cases, 14%), fish (30 cases, 9%), and seal (24 cases, 7%) were the food or beverage items associated with the largest number of laboratory confirmed botulism cases.

Botulinum neurotoxins type A (211 cases, 65%) was the toxin type most frequently identified, followed by toxin type E (82 cases, 25%) and toxin type B (22 cases, 7%). States with the largest number of reported cases were Alaska (90 cases, 28%), California (50 cases, 15%) and Ohio (40 cases, 12%). Toxin type E was identified in 93% of Alaska cases in which the toxin type was reported. Nationwide, most cases were reported between April and August (Supplementary Figure 1).

## Outbreaks of Foodborne Botulism in the United States, 2001–2017

Because of botulism’s potential to cause outbreaks through the foodborne route and the potential for severe illness for any case that occurs, a single case of botulism constitutes a public health emergency, as it may herald a larger event. From 2001 to 2017, 53 foodborne outbreaks of two or more cases were reported, and 223 (68%) of all reported foodborne botulism cases were part of an outbreak. Foodborne botulism cases are usually sporadic (single cases) or involve few cases, but large outbreaks do occur. For the purpose of this report, “large outbreak” was defined as a laboratory confirmed outbreak of foodborne botulism that affected 10 or more people. This number was based on the average number of four cases per outbreak (United States, 2001–2017). We focused on these large outbreaks to illustrate how the occurrence of botulism by the foodborne route can cause many cases of severe disease, which can strain local or regional medical resources. From 2001 to 2017, five large outbreaks of foodborne botulism were reported in the United States (Table 2; Kalluri et al., 2003; Ginsberg et al., 2007; Juliao et al., 2013; McCarty et al., 2015; McCrickard et al., 2017; Rosen et al., 2018, 2020).

**TABLE 2:** Outbreaks of laboratory confirmed botulism affecting 10 or more people, United States, 2001–2017.

Year	State	Food source	Serotype	No. of cases	References
2001	Texas	Chili	A	16	Kalluri et al., 2003
2007	Multi-state	Hot dog chili sauce	A	10	Ginsberg et al., 2007; Juliao et al., 2013
2015	Ohio	Potato salad	A	29	McCarty et al., 2015
2016	Mississippi	Pruno	A	31	McCrickard et al., 2017
2017	California	Nacho cheese	A	10	Rosen et al., 2018, 2020

### Commercially Prepared Chili in Texas, 2001

This outbreak occurred among attendees of a church dinner. Fourteen of 24 (58%) attendees who ate commercially prepared frozen chili developed signs and symptoms of botulism. Stool specimens from 8 of the 14 patients (56%) tested positive for BoNT type A. A stool sample from an attendee who ate chili and remained asymptomatic tested positive for BoNT type A, an exceedingly rare finding. An additional botulism case occurred in Dallas, in a person who did not attend the church event but had consumed chili of the same brand as the chili served at the church. BoNT type A was identified in, and *C. botulinum* type A isolated from, chili leftovers from the church supper and in the opened container of frozen chili eaten by the patient who did not attend the church event. An unopened plastic tub of the same brand of frozen chili, found in the church’s kitchen, tested positive for BoNT type A; the level of BoNT was estimated as 10,000 mouse lethal dose 50%/g (mLD_50_/g), i.e., 2 × 10^7^ mLD_50_/container. Both frozen chili packages had been purchased at the same salvage food store (discount grocery store) on sequential days. The chili served at the church event had been prepared by heating the chili without bringing it to a boil, then adding hot dogs and a different, commercially canned chili. The patient who did not attend the church event also heated the frozen chili without boiling it. Inspection of the salvage store revealed gross mishandling of foods, including opened plastic containers of food, and foods that required refrigeration or freezing but were displayed for sale at room temperature. Inspections of the chili manufacturing plant did not reveal deficiencies of production practices or product mishandling.

### Commercially Prepared Hot Dog Chili, Multi-State, 2007

This outbreak was associated with 10 cases in California (1 case), Indiana (2 cases), New Mexico (1 case), Ohio (3 cases), and Texas (3 cases). All patients developed signs and symptoms of botulism; five were treated with botulinum antitoxin. The three patients in Texas had shared a meal, which included commercially canned hot dog chili sauce. The two Indiana patients had also shared a meal, which included commercially manufactured chili of the same brand as the patients in Texas. Although the three Ohio patients were not related to each another, strong evidence indicated that they had consumed hot dog chili sauce of the same brand as the patients in Texas and Indiana. The case in California and the case in New Mexico manifested clinical signs consistent with botulism but could not be definitively linked to the implicated product through interviews or product collection from the homes. Serum and stool specimens from two of the eight patients tested positive for BoNT Type A and *C. botulinum* type A, respectively. Leftover chili mixture obtained from the home of the Indiana patients tested positive for BoNT type A and the toxin level was estimated as 4,000 mLD_50_/g. BoNT type A was also identified in leftover chili sauce collected from the home of one of the Ohio patients. No food leftovers from the cases in Texas were available for testing and an unopened can of the same product found in the patients’ home was negative for BoNT. An investigation of the cannery identified violations of canned food regulations that may have permitted spores of *C. botulinum* to survive the canning process: heating vessels used to sterilize cans were improperly maintained and operated; cooling water valves were malfunctioning; faulty alarm indicator lights and improperly calibrated temperature monitoring devices were observed. Twenty of 23 swollen cans of hot dog chili sauce collected during the investigation tested positive for BoNT type A. The manufacturer issued a nationwide recall of all products manufactured on the same production line; more than 100 million cans were recalled. No additional botulism cases were identified.

### Homemade Potato Salad in Ohio, 2015

This outbreak occurred among people who attended a church potluck meal. Twenty-nine of 77 persons (38%) who consumed potluck food had signs or symptoms consistent with botulism. Nineteen cases were laboratory confirmed, and 10 were probable cases (patients showed signs or symptoms of botulism but cases were not laboratory confirmed). One patient died of respiratory failure shortly after arriving at the hospital. Twenty-five patients were treated with botulinum antitoxin. Serum and stool specimens tested positive for BoNT type A and/or *C. botulinum* type A. Symptoms began a median of 2 days after the meal (range: 1 to 6 days). More than 50 foods were reportedly served at the event; interviews conducted among 75 participants suggested potato salad as the source. All foods served at the event had been discarded; 12 food samples were collected from the church dumpster. Six samples were positive for BoNT type A; of these, five contained potato salad and one contained macaroni and cheese that might have been cross-contaminated after being discarded. Levels of toxin in the food samples were not determined. The potato salad had been prepared with home-canned potatoes that were canned using a boiling water canner. Boiling water’s temperature is not sufficient to destroy spores of *C. botulinum* that might have been present in the raw potatoes. In addition, the canned potatoes were not heated before preparing the salad.

### Pruno (Prison Brew) in Mississippi, 2016

This outbreak occurred among inmates at a federal prison. The suspected source of BoNT was pruno, an illicit alcoholic beverage made by inmates by fermenting fruit or vegetables, sugar, water, and other ingredients. Thirty-one of 33 inmates who had consumed pruno presented with signs and symptoms of botulism. Twenty patients were treated with botulinum antitoxin. No deaths were reported. Serum and stool samples from 19 patients tested positive for BoNT type A or *C. botulinum* type A. Symptoms began a median of 3 days after first consuming pruno (range: several hours to 11 days). Reportedly, the pruno was made by combining honey, potatoes, apples, and tomato paste from a bulging can, then fermenting the mixture in a sealed plastic bag at room temperature for three to 5 days. About 20 gallons of pruno were confiscated during an investigation, but no leftovers of the batch implicated in this outbreak were found. The first two reported foodborne botulism outbreaks from pruno occurred among inmates at two prisons in California, in 2004 and 2005 (Vugia et al., 2009). Since then, four additional botulism outbreaks from pruno have been reported in the United States; the outbreak in Mississippi affected the largest number of inmates. This was the largest foodborne botulism outbreak in the United States since 1978.

### Commercially Prepared Nacho Cheese Sauce in California, 2017

Ten people were associated with an outbreak of foodborne botulism linked to nacho cheese sauce served from a dispenser at a gas station market. Symptoms began a median of 3 days after consuming cheese sauce (range: 2 to 9 days). All patients were hospitalized and were treated with botulinum antitoxin. Seven patients required mechanical ventilation and one patient died. Eight of the patients confirmed eating nacho cheese sauce at the same gas station food market; one patient died before an interview could be conducted, but had been at the gas station in the week before illness onset; the other patient worked at the gas station but did not confirm eating cheese sauce. Serum and/or stool specimens from all patients were positive for BoNT type A and/or *C. botulinum* isolates type A. A remaining pouch of nacho cheese sauce tested positive for BoNT type A and *C. botulinum* type A. Although the mechanism of contamination is unclear, inspection of the gas station indicated that the cheese in the dispenser was about 30 days past expiration date. The approximately 20 ounces of sauce found by investigators in the dispenser exhibited apparent oil separation, a temperature of 111°F, and pH of 5.95, which would allow growth of *C. botulinum* and toxin production. According to the manufacturer’s instructions, the cheese sauce should have been consumed or discarded within 5 days of placing in the dispenser, and the sauce temperature should have been maintained at 140°F. An unopened bag of cheese from different lot obtained at the gas station tested negative for BoNT. No other botulism cases associated with this commercial cheese sauce were identified.

## Public Health Preparedness and Response Approach in the United States

Every case of suspected botulism is a public health emergency, because of the potential for severe disease, and the possibility that it could be the herald of a larger outbreak from contaminated food. Therefore, public health preparedness for botulism rests most fundamentally on early clinical diagnosis by the astute clinician and immediate notification of public health authorities (Sobel, 2005).

In the United States, diagnosis of botulism, provision of botulinum antitoxin, and epidemiologic investigation are closely linked. Physicians suspecting botulism in a patient should immediately contact their state health departments’ emergency phone number (CDC, 2021). The physician will be put in touch with an expert from CDC’s Botulism Clinical Consultation Service, or, in California or Alaska, at the state health department. When indicated, CDC will urgently dispatch botulinum antitoxin from the federal stockpiles at no charge, and the state health department will facilitate collection of specimens and their testing at a specialized public health laboratory. State health departments will quickly initiate an epidemiologic investigation to identify and control the source of BoNT and determine if there are other cases (Sobel, 2005).

Since botulism can result in respiratory collapse and ventilator dependence lasting weeks to months, an outbreak involving many cases could strain or overwhelm intensive care resources locally or regionally. For example, an outbreak of 29 cases exceeded the resources of a local, modern American hospital and strained those of its surrounding metropolitan area (McCarty et al., 2015); a contaminated, widely consumed food could produce a far larger outbreak. In a mass event, worried asymptomatic persons and those with symptoms that might be due to botulism may seek medical care; the need to rapidly identify patients in danger of respiratory failure must be anticipated. In a large event, the time required to ship botulinum antitoxin, which can limit the extent of botulism-induced paralysis and prevent respiratory collapse, might result in temporary delays in administering antitoxin. Emergency preparedness planning by hospitals and local and state public health officials should incorporate consideration of such concerns into emergency planning. Clinical, laboratory diagnostic, and emergency preparedness considerations for botulism are comprehensively addressed in CDC’s botulism clinical guidelines (Sobel and Rao, 2017; Rao et al., 2021).

## Discussion

Botulism is a rare disease seen by few medical providers, which produces severe and potentially fatal illness. Botulism should always be considered a public health emergency because it can cause severe disease and potentially large outbreaks by the foodborne route; contamination of widely consumed food could cause many cases, which in turn could strain local or regional medical resources. Public health investigation and interventions to remove suspect foods can prevent additional cases and deaths. Results of investigations can help guide food manufacturers, environmental health specialists, and the general public about safe preparation and handling of foods.

## Author Contributions

All authors listed have made a substantial, direct and intellectual contribution to the work, and approved it for publication.

## Conflict of Interest

The authors declare that the research was conducted in the absence of any commercial or financial relationships that could be construed as a potential conflict of interest.

## Supplementary Material

Supplementary Figure 1 Distribution of foodborne botulism cases per month of the year, United States, 2001–2017.

## References

[B1] CDC (2011). *Botulism (Clostridium botulinum) 2011 Case Definition.* Available online at: https://wwwn.cdc.gov/nndss/conditions/botulism/case-definition/2011/2011 (accessed May, 2021).

[B2] CDC (2017). *National Botulism Surveillance, Annual Summaries.* Available online at: https://www.cdc.gov/botulism/surveillance.html (accessed May, 2021).

[B3] CDC (2021). *Botulism, Information for Health Professionals.* Available online at: https://www.cdc.gov/botulism/health-professional.html2021 (accessed May, 2021).

[B4] Ginsberg M. M. Granzow L. Teclaw R. F. Gaul L. K. Bagdure S. Cole A. (2007). Botulism associated with commercially canned chili sauce — Texas and Indiana, July 2007. *MMWR* 56 767–769. 17673898

[B5] Juliao P. C. Maslanka S. Dykes J. Gaul L. Bagdure S. Granzow-Kibiger L. (2013). National outbreak of type a foodborne botulism associated with a widely distributed commercially canned hot dog chili sauce. *Clin. Infect. Dis.* 56 376–382. 10.1093/cid/cis901 23097586 PMC4538949

[B6] Kalluri P. Crowe C. Reller M. Gaul L. Hayslett J. Barth S. (2003). An outbreak of foodborne botulism associated with food sold at a salvage store in Texas. *Clin. Infect. Dis.* 37 1490–1495. 10.1086/379326 14614672

[B7] McCarty C. L. Angelo K. Beer K. D. Cibulskas-White K. Quinn K. de Fijter S. (2015). Large outbreak of botulism associated with a church potluck meal–Ohio, 2015. *MMWR Morb. Mortal. Wkly. Rep.* 64 802–803. 10.15585/mmwr.mm6429a6 26225479 PMC4584836

[B8] McCrickard L. Marlow M. Self J. L. Watkins L. F. Chatham-Stephens K. Anderson J. (2017). Notes from the field: botulism outbreak from drinking prison-made illicit alcohol in a federal correctional facility - Mississippi, June 2016. *MMWR Morb. Mortal. Wkly. Rep.* 65 1491–1492. 10.15585/mmwr.mm6552a8 28056003

[B9] Rao A. K. Sobel J. Chatham-Stephens K. Luquez C. (2021). Clinical guidelines for diagnosis and treatment of botulism: sporadic cases and outbreaks. *MMWR* 70 1–30. 10.15585/mmwr.rr7002a1 33956777 PMC8112830

[B10] Rosen H. E. Kimura A. C. Crandall J. Poe A. Nash J. Boetzer J. (2020). Foodborne botulism outbreak associated with commercial nacho cheese sauce from a gas station market. *Clin. Infect. Dis.* 70 1695–1700. 10.1093/cid/ciz479 31247064

[B11] Rosen H. E. Kimura A. C. Mukhopadhyay R. Nash J. Boetzer J. Poe A. (2018). An outbreak of botulism associated with nacho cheese sauce from a gas station in California. *Open Forum Infect. Dis.* 5:S53. 10.1093/cid/ciz479 31247064

[B12] Sobel J. (2005). Botulism. *Clin. Infect. Dis.* 41 1167–1173. 16163636 10.1086/444507

[B13] Sobel J. Rao A. K. (2017). Making the best of the evidence: toward national clinical guidelines for botulism. *Clin. Infect. Dis.* 66(suppl. 1) S1–S3. 29293933 10.1093/cid/cix829

[B14] Vugia D. J. Mase S. R. Cole B. Stiles J. Rosenberg J. Velasquez L. (2009). Botulism from drinking pruno. *Emerg. Infect. Dis.* 15 69–71. 10.3201/eid1501.081024 19116055 PMC2660710

