# Supplementary figures and images for: Foodborne Botulism Outbreaks in the United States, 2001–2017
Source: Front Microbiol. 2021 Jul 16;12:713101. doi: 10.3389/fmicb.2021.713101 (PMC8322756; doi:10.3389/fmicb.2021.713101)

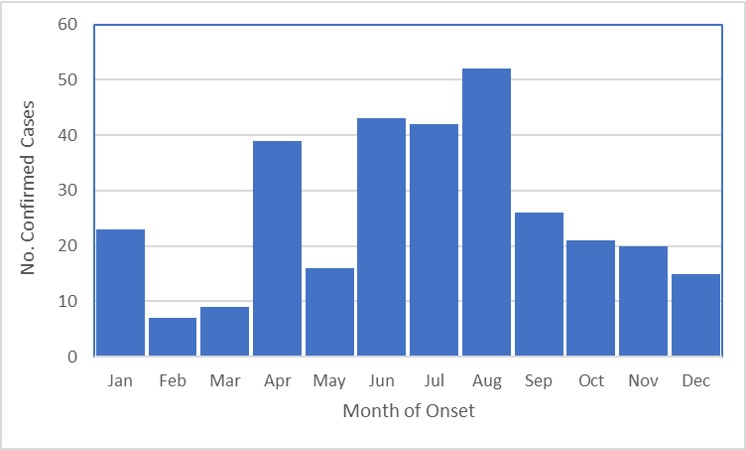

Supplement: Supplementary Figure 1 — Distribution of foodborne botulism cases per month of the year, United States, 2001–2017. [file Image_1.JPEG]
